# Comparative Effectiveness of Generic vs Brand-Name Levothyroxine in Achieving Normal Thyrotropin Levels

**DOI:** 10.1001/jamanetworkopen.2020.17645

**Published:** 2020-09-30

**Authors:** Juan P. Brito, Joseph S. Ross, Lindsey Sangaralingham, Sarah K. Dutcher, David J. Graham, Zhong Wang, Yute Wu, Xiaoxi Yao, Robert C. Smallridge, Victor Bernet, Nilay D. Shah, Kasia J. Lipska

**Affiliations:** 1 Knowledge and Evaluation Research Unit, Division of Endocrinology, Diabetes, Metabolism and Nutrition, Department of Internal Medicine, Mayo Clinic, Rochester, Minnesota; 2 Section of General Internal Medicine and the National Clinician Scholars Program, Yale School of Medicine, New Haven, Connecticut; 3 Department of Health Policy and Management, Yale School of Public Health, New Haven, Connecticut; 4 Center for Outcomes Research and Evaluation, Yale–New Haven Hospital, New Haven, Connecticut; 5 Robert D. and Patricia E. Kern Center for the Science of Health Care Delivery, Mayo Clinic, Rochester, Minnesota; 6 Office of Surveillance and Epidemiology, Center for Drug Evaluation and Research, Food and Drug Administration (FDA), Silver Spring, Maryland; 7 Office of Research and Standards, Office of Generic Drugs, Center for Drug Evaluation and Research, FDA, Silver Spring, Maryland; 8 Office of Biostatistics, Office of Translational Sciences, Center for Drug Evaluation and Research, FDA, Silver Spring, Maryland; 9 Division of Health Care Policy & Research, Mayo Clinic, Rochester, Minnesota; 10 Division of Endocrinology, Mayo Clinic, Jacksonville, Florida; 11 Optum Labs, Cambridge, Massachusetts; 12 Section of Endocrinology, Department of Internal Medicine, Yale School of Medicine, New Haven, Connecticut

## Abstract

**Importance:**

Whether the use of generic vs brand levothyroxine affects thyrotropin levels remains unclear.

**Objective:**

To compare the effectiveness of generic vs brand levothyroxine in achieving and maintaining normal thyrotropin levels among new users.

**Design, Setting, and Participants:**

This retrospective, 1:1 propensity score–matched longitudinal cohort study used the OptumLabs Data Warehouse administrative claims database linked to laboratory results from commercially insured and Medicare Advantage enrollees throughout the United States. Eligible patients were adults (aged ≥18 years) with thyrotropin levels ranging from 4.5 to 19.9 mIU/L who initiated use of generic or brand-name levothyroxine from January 1, 2008, to October 1, 2017. Data were analyzed from August 13, 2018, to October 25, 2019.

**Exposure:**

Patients received generic or brand-name levothyroxine.

**Main Outcomes and Measures:**

Proportion of patients with normal vs markedly abnormal thyrotropin levels (<0.1 or >10 mIU/L) within 3 months and with stable thyrotropin levels within 3 months after the thyrotropin value fell into the normal range.

**Results:**

A total of 17 598 patients were included (69.0% female; 74.0% White; mean [SD] age, 55.1 [16.0] years), of whom 15 299 filled generic and 2299 filled brand-name levothyroxine prescriptions during the study period. Among 4570 propensity score–matched patients (mean [SD] age, 50.3 [13.8] years; 3457 [75.6%] female; 3510 [76.8%] White), the proportion with normal thyrotropin levels within 3 months of filling levothyroxine prescriptions was similar for patients who received generic vs brand-name levothyroxine (1722 [75.4%; 95% CI, 71.9%-79.0%] vs 1757 [76.9%; 95% CI, 73.4%-80.6%]; *P* = .23), as was the proportion with markedly abnormal levels (94 [4.1%; 95% CI, 3.4%-5.0%] vs 88 [3.9%; 95% CI, 3.1%-4.7%]; *P* = .65). Among 1034 propensity score–matched patients who achieved a normal thyrotropin value within 3 months of initiation of levothyroxine, the proportion maintaining subsequent normal thyrotropin levels during the next 3 months was similar for patients receiving generic vs brand-name levothyroxine (427 [82.6%] vs 433 [83.8%]; *P* = .62).

**Conclusions and Relevance:**

Initiation of generic vs brand-name levothyroxine formulations was associated with similar rates of normal and stable thyrotropin levels. These results suggest that generic levothyroxine as initial therapy for mild thyroid dysfunction is as effective as brand-name levothyroxine.

## Key Points

**Question:**

What is the comparative effectiveness of generic vs brand-name levothyroxine in achieving normal thyrotropin levels?

**Findings:**

In a cohort study of 17 598 patients from a national administrative claims database, a similar proportion of generic vs brand-name levothyroxine users achieved target thyrotropin levels.

**Meaning:**

These findings suggest that initiation of generic or brand levothyroxine for mild thyroid dysfunction is associated with similar rates of achieving target laboratory outcomes.

## Teaser

This cohort study compares the effectiveness of generic vs brand-name levothyroxine in achieving and maintaining normal thyrotropin levels among new users.

## Introduction

Approximately 20 million Americans have overt or subclinical hypothyroidism and may require thyroid hormone replacement with levothyroxine preparations.^1,2^ In the United States, several generic and brand-name levothyroxine formulations are available for use. Generic levothyroxine preparations are less expensive and have been rated as bioequivalent by the US Food and Drug Administration (FDA) to their brand-name reference-listed drugs.^3^ However, generic levothyroxine has been less widely prescribed than other generic pharmaceutical products.^4^ Endocrinologists, in particular, are more than 3 times as likely to prescribe brand-name vs generic levothyroxine as initial therapy compared with general practitioners.^5^ In a survey of 880 members of several endocrine societies, half of the respondents indicated that they preferred brand-name to generic levothyroxine.^6^ Despite the entrenched preference for brand-name levothyroxine, it is still unclear whether brand-name levothyroxine is more effective in achieving normal and stable thyrotropin levels compared with generic levothyroxine.

To assess the comparative effectiveness of generic vs brand-name levothyroxine in patients with hypothyroidism, we used a national administrative claims database to identify patients who newly initiated use of levothyroxine. Our goal was to examine the association of generic vs brand-name levothyroxine formulation use with subsequent thyrotropin levels, the widely accepted laboratory test of thyroid status. A better understanding of the comparative effectiveness of generic compared with brand-name levothyroxine preparations may help physicians optimize their decisions about levothyroxine treatment. In turn, the findings may affect the management of hypothyroidism in millions of patients in the United States and the costs of levothyroxine use.

## Methods

### Study Design and Data Source

This cohort study adhered to the International Society for Pharmacoeconomics and Outcomes Research (ISPOR) reporting guideline for defining, reporting, and interpreting nonrandomized studies of treatment effects using secondary data sources.^7^ We conducted a retrospective analysis of deidentified administrative claims data linked with laboratory results from a large database, OptumLabs Data Warehouse, which includes privately insured patients and Medicare Advantage enrollees throughout the United States.^8^ The database contains longitudinal health information on enrollees and patients, representing a diverse mix of ages, ethnicities, and geographic regions across the United States.^9^ The health plan provides comprehensive full insurance coverage for physician, hospital, and prescription drug services and drug doses. Pharmacy claims include information on medications dispensed, including amount and dates of prescriptions. Laboratory data, available for a subset of the cohort on the basis of data sharing agreements, include test names, logical observation identifier names, and test results. Study data were accessed using techniques compliant with the Health Insurance Portability and Accountability Act of 1996. Because this study involved analysis of preexisting, deidentified data, the Mayo Clinic institutional review board declared it exempt from board approval and informed consent.

### Study Population

**Figure 1.**
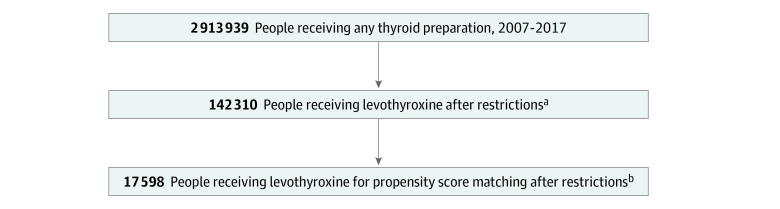
Study Population ^a^The study population was limited to those 18 years or older with coverage 365 days before and 90 days after prescription date, prescriptions starting after 2008, prescriptions for levothyroxine, and thyrotropin measurements at baseline and 3 months. ^b^Patients with thyroid cancer, hypopituitarism, thyroiditis, or hyperthyroidism, who were pregnant or nonadherent, or who switched formulations were excluded.

We identified 2 cohorts. Cohort 1 consisted of adult patients (aged ≥18 years) who newly filled prescriptions for either generic or brand-name levothyroxine preparations from January 1, 2008, to October 1, 2017. Patients were required to have at least 365 days of continuous medical and pharmacy benefits coverage before treatment initiation (index date) and 90 days after initiation. We applied a number of cohort exclusions. We excluded patients who filled any thyroid preparation within the 365 days before the index data (ie, only new users were included). We also excluded patients who switched from generic to brand-name or from brand-name to generic levothyroxine within 90 days (3 months) of treatment initiation, and patients who used other forms of thyroid hormone replacement therapy at any point during follow-up, including thyroid extracts or triiodothyronine therapy (liothyronine, thyroid desiccated/extracts, Cytomel [Pfizer Inc], Armour Thyroid [Allergan], and Nature-Thyroid [RLC Labs]). We also excluded patients who were not adherent to levothyroxine therapy. Adherence was measured by the proportion of days covered at 90 days, and patients were considered adherent if the proportion of days covered was greater than 80.^10,11^ We also excluded patients taking any levothyroxine dose exceeding 200 μg/d owing to difficulty assessing the full prescribed dose (maximum dose in a single tablet is 200 μg) and because doses greater than 200 μg might suggest levothyroxine malabsorption issues. To assess thyroid status, we further limited our study to patients who had linked thyrotropin results at both baseline (within 90 days before drug initiation) and follow-up (within 6-12 weeks after initiation). Only patients with baseline thyrotropin values ranging from 4.5 to 19.9 mIU/L were included because those with thyrotropin values greater than 20.0 mIU/L may take longer than 3 months to achieve the normal range of levels. Finally, we excluded patients with conditions that require specific thyrotropin targets that may fall outside of the reference range, such as pregnancy, thyroid cancer, and hypopituitarism, or patients exposed to medications known to affect thyroid hormone levels during the baseline and follow-up periods (a list of medications and *International Classification of Diseases* codes for excluded conditions is found in eTable 1 in the Supplement).^12^ Enrollment of the study population is depicted in Figure 1.

Cohort 2 was a subset of cohort 1. We included only patients from cohort 1 who had a normal follow-up thyrotropin level within 3 months and a subsequent follow-up thyrotropin level measured 6 to 12 weeks after the thyrotropin test with normal findings. Given that these patients had a previous normal thyrotropin value, the individuals were likely to be receiving the same dose of levothyroxine. We also excluded patients who switched from generic to brand-name or from brand-name to generic levothyroxine within 180 days (6 months) of treatment initiation.

Baseline patient characteristics included age, sex, race/ethnicity, census region, physician specialty (general vs endocrinology vs other specialist), year of index prescription, health plan type (commercial vs Medicare Advantage), Charlson comorbidity index score^9^ (estimated using *International Classification of Diseases, Ninth Revision, Clinical Modification,* or *International Statistical Classification of Diseases and Related Health Problems, Tenth Revision*, diagnosis codes included in administrative claims), thyrotropin level, levothyroxine dose (calculated based on fill data), conditions that may increase the risk of levothyroxine malabsorption (inflammatory bowel disease, anemia as surrogate of iron use), and use of estrogen within 3 months of index date.

### Exposures

We characterized whether the index pharmacy fill was for a generic or a brand-name thyroid hormone drug. We used First Databank to categorize each fill as brand-name or generic. First Databank categorizes pharmacy products as generic if they are sold under a generic pharmacy label, which includes authorized generic products. The brand-name products included Synthroid (AbbVie; 2042 [88.8%]), Levoxyl (Pfizer; 178 [7.7%]), and others (79 [3.4%]). The generic manufacturers included Mylan Pharmaceuticals (7930 [51.8%]), Sandoz AG (1733 [11.3%]), and Lannett Company, Inc (5636 [36.8%]).

### Study Outcomes

**Figure 2.**
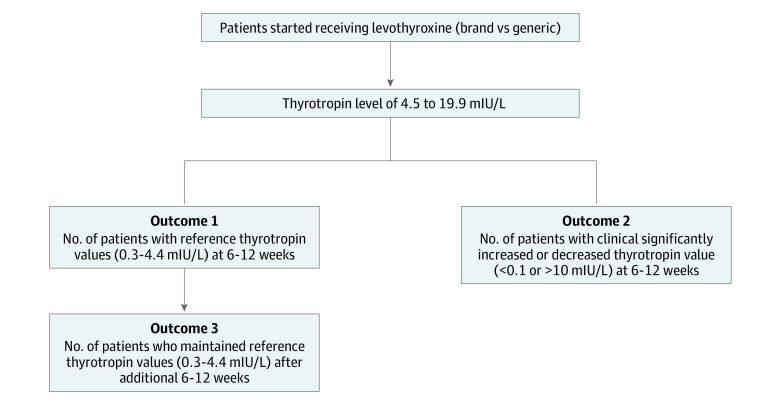
Study Outcomes

We examined the effectiveness of levothyroxine based on attained thyrotropin levels measured in the outpatient setting. For assessment of effectiveness, we examined the proportion of individuals who initiated use of either generic or brand-name levothyroxine and who attained a normal thyrotropin level within 3 months, clinically meaningful abnormal thyrotropin level within 3 months, and stable thyrotropin level(s) within 3 months after thyrotropin fell into the normal range (Figure 2).

We defined a normal thyrotropin level as ranging from 0.3 to 4.4 mIU/L, a range that includes as much as 95% of the healthy population.^13^ Given that thyrotropin values are only reliable as treatment markers at least 6 weeks after initiation of therapy,^12^ we used only the first available thyrotropin value from 6 weeks to 3 months after initiation of therapy to assess treatment effectiveness. Using thyrotropin values beyond 3 months of therapy may not be an accurate surrogate for effectiveness because the values might reflect changes in doses rather than the comparative effectiveness between brand-name and generic levothyroxine. Moreover, levothyroxine dose adjustments may not be reliably obtained retrospectively from the OptumLabs Data Warehouse, because physician-directed medication adjustments may involve pill splitting and alternate-day dosing. Abnormal thyrotropin values were categorized as clinically meaningful when thyrotropin levels were suppressed (<0.1 mIU/L) or elevated (>10 mIU/L). We defined stable thyrotropin levels as a normal thyrotropin level within 3 months after thyrotropin levels fell into the normal range. When there were multiple thyrotropin level measurements during this period of time, we used the one closest to the first thyrotropin measurement (Figure 2).

### Statistical Analysis

Data were analyzed from August 13, 2018, to October 25, 2019. We used propensity scores to minimize confounding. Patients receiving generic levothyroxine were matched 1:1 with patients receiving brand-name levothyroxine using nearest-neighbor matching with a caliper of 0.2. We developed a propensity score model with the binary outcome of initiating treatment with brand-name levothyroxine. The model included demographics, comorbidities, and baseline thyrotropin values shown in Table 1 (cohort 1) and Table 2 (cohort 2). Variables within these models were selected based on clinical relevance and evidence from prior studies. For each cohort, we evaluated the balance among the treatment groups by comparing standardized mean differences of baseline covariates between the groups. A baseline characteristic was considered balanced if the maximum standardized mean difference was less than 10%. Using this matched cohort, we then tested for differences between patients treated with generic vs brand-name levothyroxine using χ^2^ tests for categorical outcomes. We used SAS software, version 9.4 (SAS Institute, Inc), for all statistical analyses. We considered a 2-sided *P* < .05 to be statistically significant.

**Table 1.** Baseline Characteristics of Cohort 1^a^

Characteristic	Prematch			1:1 match^b^		
	Levothyroxine group		SMD^c^	Levothyroxine group		SMD^c^
	Generic (n = 15 299)	Brand-name (n = 2299)		Generic (n = 2285)	Brand-name (n = 2285)	
Initiating dose, μg/d						
≤50	13 245 (86.6)	1875 (81.6)	−0.14	1899 (83.1)	1865 (81.6)	−0.04
51-100	1799 (11.8)	357 (15.5)	0.11	334 (14.6)	355 (15.5)	0.03
101-200	255 (1.7)	67 (2.9)	0.08	52 (2.3)	65 (2.8)	0.03
Thyrotropin level, mIU/L						
4.5-9.9	13 340 (87.2)	2027 (88.2)	0.03	2041 (89.3)	2015 (88.2)	−0.03
10.0-19.9	1959 (12.8)	272 (11.8)	−0.03	244 (10.7)	270 (11.8)	0.03
Mean (SD)	7.0 (2.8)	6.9 (2.8)	0.04	6.8 (2.6)	6.9 (2.8)	−0.04
Median (IQR)	6.1 (5.2-7.8)	6.0 (5.1-7.7)	NA	5.9 (5.1-7.5)	6.0 (5.1-7.6)	NA
Age, y						
Mean (SD)	55.8 (16.2)	50.4 (13.7)	0.36	50.2 (13.8)	50.4 (13.7)	−0.02
Median (IQR)	56.0 (45.0-68.0)	51.0 (41.0-60.0)	NA	50.0 (41.0-60.0)	51.0 (41.0-60.0)	NA
Sex						
Female	10 410 (68.0)	1726 (75.1)	0.16	1741 (76.2)	1716 (75.1)	−0.03
Male	4889 (32.0)	573 (24.9)	−0.16	544 (23.8)	569 (24.9)	0.03
Race/ethnicity						
Asian	659 (4.3)	107 (4.7)	0.02	92 (4.0)	104 (4.6)	0.03
Black	1281 (8.4)	177 (7.7)	−0.03	164 (7.2)	173 (7.6)	0.02
Hispanic	1572 (10.3)	206 (9.0)	−0.04	187 (8.2)	206 (9.0)	0.03
White	11 289 (73.8)	1734 (75.4)	0.04	1783 (78.0)	1727 (75.6)	−0.06
Unknown	498 (3.3)	75 (3.3)	0	59 (2.6)	75 (3.3)	0.04
Insurance plan						
Commercial	10 527 (68.8)	2032 (88.4)	0.49	2020 (88.4)	2018 (88.3)	0
Medicare Advantage	4772 (31.2)	267 (11.6)	−0.49	265 (11.6)	267 (11.7)	0
Census region						
Midwest	1961 (12.8)	212 (9.2)	−0.12	200 (8.8)	212 (9.3)	0.02
Northeast	1500 (9.8)	211 (9.2)	−0.02	218 (9.5)	209 (9.1)	−0.01
South	9024 (59.0)	1573 (68.4)	0.2	1552 (67.9)	1562 (68.4)	0.01
West	2814 (18.4)	303 (13.2)	−0.15	315 (13.8)	302 (13.2)	−0.02
Charlson comorbidity index score at baseline						
Mean (SD)	1.1 (1.9)	0.7 (1.4)	0.24	0.6 (1.2)	0.7 (1.4)	−0.08
Median (IQR)	0 (0-1.0)	0 (0-1.0)	NA	0 (0-1.0)	0 (0-1.0)	NA
Conditions affecting levothyroxine absorption						
Inflammatory bowel disease	109 (0.7)	22 (1.0)	0.03	13 (0.6)	21 (0.9)	0.04
Anemia	892 (5.8)	120 (5.2)	−0.03	108 (4.7)	120 (5.3)	0.03
Estrogen use 90 d prior	1648 (10.8)	377 (16.4)	0.16	342 (15.0)	375 (16.4)	0.04
Prescribing physician specialty						
Endocrinologist	719 (4.7)	438 (19.1)	0.46	414 (18.1)	425 (18.6)	0.01
General	11 345 (74.2)	1507 (65.6)	−0.19	1523 (66.7)	1507 (66.0)	−0.01
Missing/other	3235 (21.1)	354 (15.4)	−0.15	348 (15.2)	353 (15.4)	0.01

Abbreviations: IQR, interquartile range; NA, not applicable; SMD, standardized mean difference.

^a^ Includes adult patients who newly filled generic or brand-name levothyroxine preparations from January 1, 2008, to October 1, 2017. Unless otherwise indicated, data are expressed as number (percentage) of patients. Percentages have been rounded and may not total 100.

^b^ Also matched on year of index prescription.

^c^ A baseline characteristic was considered balanced if the maximum SMD was less than 0.10.

**Table 2.** Baseline Characteristics of Cohort 2^a^

Characteristic	Prematch			1:1 match^b^		
	Levothyroxine group		SMD^c^	Levothyroxine group		SMD^c^
	Generic (n = 2949)	Brand-name (n = 534)		Generic (n = 517)	Brand-name (n = 517)	
Age, y						
Mean (SD)	55.8 (16.0)	50.1 (13.4)	0.39	50.3 (14.1)	50.3 (13.4)	0
Median (IQR)	56 (45.0-68.0)	50 (41.0-60.0)	NA	50 (41.0-60.0)	50 (41.0-60.0)	NA
Initiating dose, μg/d						
≤50	2615 (88.7)	433 (81.1)	−0.21	433 (83.8)	423 (81.8)	−0.05
51-200	334 (11.3)	101 (18.9)	0.21	94 (18.2)	84 (16.2)	−0.05
Sex						
Female	2072 (70.3)	419 (78.5)	0.19	401 (77.6)	406 (78.5)	0.02
Male	877 (29.7)	115 (21.5)	−0.19	116 (22.4)	111 (21.5)	−0.02
Race/ethnicity						
Asian	123 (4.2)	27 (5.1)	0.04	28 (5.4)	24 (4.6)	−0.04
Black	244 (8.3)	29 (5.4)	−0.11	25 (4.8)	29 (5.6)	0.04
Hispanic	302 (10.2)	49 (9.2)	−0.03	38 (7.4)	46 (8.9)	0.05
Unknown	96 (3.3)	20 (3.7)	0.02	18 (3.5)	20 (3.9)	0.02
White	2184 (74.1)	409 (76.6)	0.06	408 (78.9)	398 (77.0)	−0.05
Insurance plan						
Commercial	1991 (67.5)	470 (88.0)	0.51	442 (85.5)	453 (87.6)	0.06
Medicare Advantage	958 (32.5)	64 (12.0)	−0.51	75 (14.5)	64 (12.4)	−0.06
Census region						
Midwest	339 (11.5)	43 (8.1)	−0.11	35 (6.8)	43 (8.3)	0.06
Northwest	315 (10.7)	45 (8.4)	−0.08	33 (6.4)	44 (8.5)	0.08
South	1809 (61.3)	375 (70.2)	0.19	377 (72.9)	361 (69.8)	−0.07
West	486 (16.5)	71 (13.3)	−0.09	72 (13.9)	69 (13.3)	−0.02
Charlson comorbidity index score						
Mean (SD)	1.2 (2.0)	0.7 (1.3)	0.30	0.7 (1.2)	0.7 (1.3)	0
Median (IQR)	0 (0-2.0)	0 (0-1.0)	NA	0 (0-1.0)	0 (0-1.0)	NA
Conditions affecting levothyroxine absorption^d^						
Estrogen use 90 d prior	356 (12.1)	75 (14.0)	0.06	65 (12.6)	72 (13.9)	0.04
Prescribing physician specialty						
Endocrinologist	217 (7.4)	136 (25.5)	0.50	113 (21.9)	121 (23.4)	0.04
General	2053 (69.6)	322 (60.3)	−0.20	332 (64.2)	320 (61.9)	−0.05
Missing/other	679 (23.0)	76 (14.2)	−0.23	72 (13.9)	76 (14.7)	0.02

Abbreviations: IQR, interquartile range; NA, not applicable; SMD, standardized mean difference.

^a^ Includes patients from cohort 1 who had a normal follow-up thyrotropin level within 3 months and who had a subsequent follow-up thyrotropin level measurement 6 to 12 weeks after the normal test result. Unless otherwise indicated, data are expressed as number (percentage) of patients. Percentages have been rounded and may not total 100.

^b^ Also matched on year of index prescription.

^c^ A baseline characteristic was considered balanced if the maximum SMD was less than 0.10.

^d^ Cell size too small for celiac, inflammatory bowel disease, anemia to include.

The comparative effectiveness of generic and brand-name levothyroxine among new users could be influenced by the presence of endogenous production of thyroid hormone (patients with a functional thyroid gland). Therefore, we examined whether outcomes differed among patients with and without endogenous thyroid hormone production. We categorized patients as not having endogenous thyroid production if they had a history of total thyroidectomy or if they received thyroid hormone doses greater than 100 μg/d. Because the mean full replacement dose of levothyroxine in adults is approximately 1.6 μg/kg per day, a dose of greater than 100 μg/d is likely to reflect full replacement.

### Sensitivity Analysis

Propensity score matching shrinks the sample to produce groups of the same size. Therefore, we used an alternative propensity score method, inverse probability of treatment weighting (IPTW), to preserve the data lost during matching to confirm our findings. Inverse probability of treatment weighting is an alternative method of incorporating propensity scores to isolate treatment effects used in observational studies. Contrary to propensity score matching, in which the resulting conditional model for the outcomes is fitted though matching, IPTW uses weighting.^14,15^

## Results

### Normal and Clinically Abnormal Thyrotropin Values

The characteristics of the 17 598 patients (5462 male [31.0%] and 12 136 female [69.0%]; 13 023 [74.0%] White; mean [SD] age, 55.1 [16.0] years) who filled levothyroxine prescriptions for the first time from 2008 to 2017 (cohort 1) are shown in Table 1. Most patients (15 299 [87.0%]) used generic levothyroxine, and the most commonly filled levothyroxine dose was no greater than 50 μg/d (15 120 [85.9%]). Patients who received generic levothyroxine were older (mean [SD] age, 55.8 [16.2] vs 50.4 [13.7] years), had a higher mean (SD) baseline Charlson comorbidity index score (1.1 [1.9] vs 0.7 [1.4]), were less likely to live in the South census region (9024 [59.0%] vs 1573 [68.4%]), were less likely to use estrogen (1648 [10.8%] vs 377 [16.4%]), and were more likely to receive their index prescription from a general practitioner (11 345 [74.2%] vs 1507 [65.6%]) compared with patients prescribed brand-name levothyroxine. After 1:1 propensity score matching, no significant differences were apparent between generic and brand-name levothyroxine groups with respect to all of the measured variables (Table 1).

Among 4570 propensity score–matched patients (mean [SD] age, 50.3 [13.8] years; 3457 female [75.6%] and 1113 male [24.4%]; 3510 White [76.8%]), the mean (SD) time from index date to the first thyrotropin level measurement was 57.71 (12.40) days (median, 56 [interquartile range, 47-66] days) among patients who filled generic levothyroxine and 56.87 (12.38) days (median, 55 [interquartile range, 46-65] days) among patients who filled brand-name levothyroxine prescriptions. Among matched pairs of patients, the proportion of patients who achieved a normal thyrotropin value within 3 months of filling their first levothyroxine prescription was similar for patients who received generic vs brand-name levothyroxine (1722 [75.4%; 95% CI, 71.9%-79.0%] vs 1757 [76.9%; 95% CI, 73.4%-80.6%]; *P* = .23). Among these matched pairs of patients, 94 (4.1%; 95% CI, 3.4%-5.0%) and 88 (3.9%; 95% CI, 3.1%-4.7%) had a clinically meaningful abnormal thyrotropin value of less than 0.1 mIU/L or greater than 10 mIU/L in the generic and brand-name cohorts, respectively (*P* = .65).

### Patients Who Maintained Normal Thyrotropin Values

A total of 3483 patients from cohort 1 had a normal follow-up thyrotropin level within 3 months and underwent subsequent follow-up thyrotropin level measurement 6 to 12 weeks after the normal thyrotropin test result. Similar to the baseline characteristics of patients in cohort 1, most patients used generic levothyroxine (2949 [84.6%]), and most used a levothyroxine dose of no more than 50 μg/d (3048 [87.5%]). After 1:1 propensity score matching, no significant differences were apparent between generic and brand-name levothyroxine groups with respect to all of the measured variables (Table 2). Among the matched cohort of 1034 patients who achieved a normal thyrotropin value within 3 months after initiation of levothyroxine therapy, the proportion of patients who had a subsequent normal thyrotropin value within the next 3 months was similar for patients using generic vs brand-name levothyroxine (427 [82.6%] vs 433 [83.8%]; *P* = .62).

### Subgroup Analyses

Within cohort 1, only 30 (0.7%) of patients taking levothyroxine had a history of total thyroidectomy in the year before the index date; thus, a subgroup analysis on this subset of patients was not possible. An analysis of the subset of patients taking thyroid hormone doses greater than 100 μg/d (n = 117) showed that normal thyrotropin values were attained in 25 of 52 (48.1%) vs 42 of 65 (64.6%) of generic vs brand-name levothyroxine users, respectively (*P* = .07). Within cohort 2, a subgroup analysis was not possible because of the low number of patients requiring doses of at least 100 μg/d.

### Sensitivity Analyses

Results using IPTW were consistent with the results of propensity score–matching analysis. Using the entire population with IPTW analyses (eTable 2 in the Supplement), the proportion of patients who achieved a normal thyrotropin value within 3 months of filling their first levothyroxine prescription was similar for patients who received generic vs brand-name levothyroxine (11 153 [72.9%; 95% CI, 71.3%-74.0%] vs 1733 [75.4%; 95% CI, 73.4%-80.5%]; *P* = .07) (eTable 3 in the Supplement). Furthermore, among patients who achieved a normal thyrotropin value within 3 months after initiation of levothyroxine therapy, the proportion who had a subsequent normal thyrotropin value within the following 3 months was similar for patients receiving generic vs brand-name levothyroxine (84.1% vs 83.3%; *P* = .74).

## Discussion

For adults with mild forms of thyroid dysfunction, defined as thyrotropin levels less than 20 mIU/L before initiation of treatment, consistent use of generic or brand-name levothyroxine formulations is associated with similar rates of achieving normal and stable thyrotropin levels, that is, laboratory-based outcomes that are considered clinically important. During the last decade, one-quarter of all levothyroxine prescriptions filled were still for brand-name levothyroxine products.^5^ Although the use of generic levothyroxine has increased over time in the United States, it continues to be relatively low compared with the use of other generic medications. For example, prior studies^4^ have demonstrated that when both generic and brand-name options are available, generic drugs are generally selected 97% of the time compared with the brand-name products.

One driver of the continued preference for brand-name levothyroxine may be physicians’ skepticism about the equivalent effectiveness of generic compared with brand-name levothyroxine. After the FDA approved the use of several generic levothyroxine products, 3 endocrine professional societies released a joint position statement in 2004 raising questions about the FDA’s method for determining bioequivalence.^16^ The position statement specifically pointed out that the FDA’s method does not consider the role of endogenously produced hormone and does not rely on the thyrotropin level. They further implied that these methodological flaws, along with the fact that levothyroxine has a relatively narrow therapeutic index, may lead to a significant difference in bioequivalence between generic and brand-name levothyroxine. In turn, this difference might affect thyrotropin levels and associated clinical outcomes.^16^ In 2007, the FDA, in response to concerns expressed about levothyroxine products by health care professionals and patients, tightened the potency specifications for levothyroxine to meet a 95% to 105% potency specification until their expiration date.^17^

Despite these changes, the concerns initially raised in 2004 by endocrine societies have continued to affect endocrine guideline recommendations and practice. For instance, the 2014 American Thyroid Association guideline specifically recommends avoidance of switches between levothyroxine products.^12^ To maintain the same preparation, the guideline recommends that physicians either prescribe the same identifiable formulation of generic levothyroxine or a specific brand-name levothyroxine. The latter strategy is logistically easier, as it avoids substitution for another levothyroxine preparation at the level of the pharmacy, which is common when a generic prescription is used and can occur without physician approval.

Our results should reassure physicians and patients that generic levothyroxine as initial therapy for mild thyroid dysfunction is as effective as brand-name levothyroxine. Moreover, prescribing generic compared with brand-name levothyroxine may offer substantial financial benefits to patients and to the health care system. For instance, information available from the OptumLabs Data Warehouse shows that in 2017 the mean 30-day out-of-pocket cost was $6.20 for generic levothyroxine and $28.65 for brand-name levothyroxine across commercial, Medicare Advantage, and Medicare Part D insurance. However, our results do not address the potential effect of switches, either purposeful or passive, between formulations. These switches may occur between brand-name and generic products or between products (either brand-name or generic) made by different levothyroxine manufacturers. Thus, our results do not directly address the issue of interchangeability of levothyroxine formulations. Additional research should compare the effectiveness of levothyroxine products among patients who switch therapy during the course of their treatment.

Our study included patients with baseline thyrotropin values ranging from 4.5 to 19.9 mIU/L, which reflect mild thyroid dysfunction. The most commonly prescribed levothyroxine dose in our cohort was no greater than 50 μg/d, which is unlikely to be a full thyroid hormone replacement dose. Thus, patients in our study likely represent a cohort with some endogenous thyroid hormone production. However, our results are likely generalizable to most patients in the United States who initiate levothyroxine therapy; most patients have partial thyroid dysfunction (eg, subclinical hypothyroidism) and may be able to maintain some endogenous thyroid hormone production.^18^

Patients with endogenous levothyroxine production might retain some ability to regulate thyroid hormone levels despite thyroid hormone replacement therapy. As a result, patients with residual endogenous levothyroxine production may have different thyrotropin values than patients without any endogenous levothyroxine receiving the same levothyroxine dose or formulation.^8,9^ In our analysis, based on a small subset of patients, the proportion of patients achieving normal thyrotropin values within 3 months was higher among patients who initiated brand vs generic levothyroxine when doses greater than 100 μg/d were prescribed, but these findings were not statistically significant. In a small crossover study that randomized children to 8 weeks of brand-name or generic levothyroxine therapy,^19^ serum thyrotropin concentrations were significantly lower when patients used brand-name levothyroxine compared with the same AB-rated generic levothyroxine (Sandoz AG) but only in the subgroup of 20 patients with acquired hypothyroidism (ie, in those with no endogenous levothyroxine production). Clearly, more research is needed to understand the comparative effectiveness and safety of brand-name vs generic levothyroxine in patients without remaining endogenous thyroid function.

### Limitations

Our study has several limitations that need to be considered. Given the observational nature of our study, residual confounding may still exist. As a result, unmeasured factors may have influenced our findings, and patients filling generic levothyroxine may be different from those filling brand levothyroxine prescriptions; these differences may have influenced the study outcomes. For instance, we could not account for all the factors that might affect gastrointestinal tract absorption of levothyroxine, such as concomitant intake of over-the-counter calcium preparations.^20^ Our primary measure for the assessment of baseline thyroid hormone level status and for the laboratory outcomes was based on a thyrotropin level and not free thyroxine. We preferred this approach, given that thyrotropin is the most sensitive test to assess response to therapy.^12^ We could not reliably ascertain levothyroxine dose adjustments from the OptumLabs Data Warehouse because physician-directed medication adjustments may involve pill cutting and alternate-day dosing. Finally, our study sample includes mostly patients with commercial health insurance; the applicability of study findings to underinsured populations is unclear.

## Conclusions

This cohort study of the comparative effectiveness of generic vs brand-name levothyroxine found that for adults with mild forms of thyroid dysfunction, consistent use of either the generic or brand-name formulations was associated with similar rates of achieving normal and stable thyrotropin levels. Further research needs to clarify whether these findings are consistent among patients with no or little endogenous thyroid hormone production and whether switching between formulation affects these outcomes.

## Notes

Supplement. **eTable 1.** List of Medications Assessed During the Baseline and Follow-up Periods and List of *ICD* Codes Used to Exclude Patients **eTable 2.** Baseline Characteristics of Cohort 1 **eTable 3.** Cohort 1 Results of IPTW
