# Supplementary material for: Comparative Effectiveness of Generic vs Brand-Name Levothyroxine in Achieving Normal Thyrotropin Levels
Source: JAMA Netw Open. 2020 Sep 30;3(9):e2017645. doi: 10.1001/jamanetworkopen.2020.17645 (PMC7527873; doi:10.1001/jamanetworkopen.2020.17645)
Supplement: Supplement. — eTable 1. List of Medications Assessed During the Baseline and Follow-up Periods and List of ICD Codes Used to Exclude Patients eTable 2. Baseline Characteristics of Cohort 1 eTable 3. Cohort 1 Results of IPTW [file jamanetwopen-e2017645-s001.pdf]

Supplementary Online Content

Brito JP, Ross JS, Sangaralingham L, et al. Comparative effectiveness of generic vs brand-name levothyroxine in achieving normal thyrotropin levels. *JAMA Netw Open*. 2020;3(9):e2017645. doi:10.1001/jamanetworkopen.2020.17645

eTable 1. List of Medications Assessed During the Baseline and Follow-up Periods and List of *ICD* Codes Used to Exclude Patients

eTable 2. Baseline Characteristics of Cohort 1

eTable 3. Cohort 1 Results of IPTW

This supplementary material has been provided by the authors to give readers additional information about their work.

©2020 Brito JP et al. *JAMA Network Open*.

eTable 1. List of Medications Assessed During the Baseline and Follow-up Periods and List of *ICD* Codes Used to Exclude Patients

PTU, Propylthiouracil, 6-N Propylthiouracil, Methimazole, Felimazole, Northyx, Tapazole, Thiamazole, Carbimazole, Benzylthiouracil, Methylthiouracil), lithium carbonate, amiodarone hydrochloride, phenytoin, interferon alfa, interleukin 2, gefitinib, erlotinib, sorafenib, sunitinib, dasatinib and lenvatinib, imatinib, cabozantinib, or vandetanib.

Pregnancy: V22X, V23X, V24X, V27X, Z34X, O09X, Z3AX
Hypopituitarism: 2537, 2532, E230, E893
Thyroid cancer: 193X, C73X

eTable 2. Baseline Characteristics of Cohort 1

	Pre-**IPTW**			After **IPTW**		
	Generic L-thyroxine (N=15,299)	Brand L-thyroxine (N=2,299)	SD	Generic L-thyroxine (N=15,299)	Brand L-thyroxine (N=2,299)	SD
**Initiating Dose (mcg)**						
<= 50	86.6%	81.6%	0.14	85.9%	85.6%	0.01
51 to 100	11.8%	15.5%	0.11	12.3%	12.4%	0.00
101 to 200	1.7%	2.9%	0.08	1.8%	2.0%	0.01
**TSH Category Baseline (mIU/L)**						
4.5 to 9.9	87.2%	88.2%	0.03	87.3%	87.4%	0.00
10-19.9	12.8%	11.8%	0.03	12.7%	12.6%	0.00
**TSH Baseline (mIU/L)**						
Mean (SD)	7.0 (2.8)	6.9 (2.8)	0.04	7.0 (2.77)	7.0 (2.90)	0.00
**Age (years)**						
Mean (SD)	55.8 (16.2)	50.4 (13.7)	0.36	55.1 (16)	54.0 (15.6)	0.02
**Sex**						
Female	68.0%	75.1%	0.16	69.0%	72.0%	0.04
Male	32.0%	24.9%	0.16	31.0%	29.0%	0.04
**Race/Ethnicity**						
Asian	4.3%	4.7%	0.02	4.3%	4.1%	0.01
Black	8.4%	7.7%	0.03	8.3%	8.5%	0.01
Hispanic	10.3%	9.0%	0.04	10.1%	10.0%	0.00
White	73.8%	75.4%	0.04	74.0	74.3%	0.01

©2020 Brito JP et al. *JAMA Network Open*.

Unknown	3.3%	3.3%	0	3.3%	3.1%	0.01
**Health Plan**						
Commercial	68.8%	88.4%	0.49	71.4%	75.2%	0.08
Medicare Advantage	31.2%	11.6%	0.49	28.6%	24.8%	0.08
**Census Region**						
Midwest	12.8%	9.2%	0.12	12.3%	11.3%	0.03
Northeast	9.8%	9.2%	0.02	9.7%	9.8%	0.00
South	59.0%	68.5%	0.20	60.3%	60.7%	0.01
West	18.4%	13.1%	0.15	17.7%	18.2%	0.01
**Charlson Baseline**						
Mean (SD)	1.1 (1.9)	0.7 (1.4)	0.24	1.0 (1.9)	1.0 (1.8)	0.03
**Conditions Affecting L-thyroxine Absorption**						
Inflammatory bowel disease	0.7%	1.0%	0.03	1.0%	1.0%	0.02
Anemia	5.8%	5.2%	-0.03	6.0%	6.0%	0.03
Estrogen Use (90 days prior)	10.8%	16.4%	0.16	11.0%	12.0%	0.00
**Prescribing Provider Specialty**						
Endocrinologist	4.7%	19.1%	0.46	6.6%	6.8%	0.01
General Provider	74.2%	65.6%	0.19	73.0%	74.3%	0.03
Missing/Other	21.1%	15.4%	0.15	20.4%	18.9%	0.04

Includes adult patients who newly filled either generic or brand L-thyroxine preparations between January 1, 2008 and October 1, 2017.

eTable 3. Cohort 1 Results of IPTW

	Generic L-thyroxine (N=15,299)	Brand L-thyroxine (N=2,299)	p-value
TSH Category Result			
Normal (0.3 to 4.4 mIU/L)	72.9% (71.3, 74.0)	75.4% (73.4, 80.5)	0.07

TSH levels within 3 months of initiation of brand vs generic L-thyroxine among weighted population of patients with abnormal baseline TSH levels (4.5 to 19.9 units)

©2020 Brito JP et al. *JAMA Network Open*.
